# Differences between men and women with nonradiographic axial spondyloarthritis: clinical characteristics and treatment effectiveness in a real-life prospective cohort

**DOI:** 10.1186/s13075-020-02337-2

**Published:** 2020-10-09

**Authors:** Regula Neuenschwander, Monika Hebeisen, Raphael Micheroli, Kristina Bürki, Pascale Exer, Karin Niedermann, Michael J. Nissen, Almut Scherer, Adrian Ciurea

**Affiliations:** 1 Department of Rheumatology, Zurich University Hospital, Gloriastrasse 25, CH-8091 Zurich, Switzerland; 2 Swiss Clinical Quality Management Foundation, Statistics Group, Zurich, Switzerland; 3 Rheumatology Practice, Basel, Switzerland; 4 School of Health Professions, Institute of Physiotherapy, Zurich University of Applied Sciences, Winterthur, Switzerland; 5 Department of Rheumatology, University Hospital, Geneva, Switzerland

**Keywords:** Axial spondyloarthritis, Nonradiographic axial spondyloarthritis, Gender, TNF inhibition

## Abstract

**Background:**

Sex differences with regard to clinical manifestations and response to tumor necrosis factor inhibitors (TNFi) have been delineated for the radiographic form of axial spondyloarthritis (axSpA). More limited evidence for a differential effectiveness of treatment in genders exists for the nonradiographic disease state (nr-axSpA). The aim of the study was to compare demographics, clinical parameters, and response to TNFi in women versus men with nr-axSpA.

**Methods:**

We compared disease characteristics of 264 women and 231 men with nr-axSpA at inclusion in the prospective Swiss Clinical Quality Management Cohort. Response to a first TNFi was assessed in 85 women and 78 men without diagnosed co-morbid fibromyalgia. The primary outcome was the proportion of patients achieving the 40% improvement in the Assessment of SpondyloArthritis international Society criteria (ASAS40) at 1 year. Additional response outcomes were evaluated as secondary outcomes. Patients having discontinued TNFi were considered non-responders. Logistic regression analyses were adjusted for baseline differences, which might potentially mediate the effect of sex on treatment response.

**Results:**

Compared to men, women had a longer diagnostic delay, a higher level of perceived disease activity, and more enthesitis and were in a lower percentage HLA-B27 positive. An ASAS40 response was achieved by 17% of women and 38% of men (OR 0.34; 95% CI 0.12, 0.93; *p* = 0.02). A significantly lower response rate in women was confirmed in the adjusted analysis (OR 0.19; 95% CI 0.05, 0.62; *p* = 0.009) as well as for the other outcomes assessed.

**Conclusion:**

Despite only few sex differences in patient characteristics in nr-axSpA, response rates to TNFi are significantly lower in women than in men.

## Background

With the introduction of the Assessment of SpondyloArthritis international Society (ASAS) classification criteria [1], patients with axial spondyloarthritis (axSpA) can be classified as having radiographic or nonradiographic disease (r-axSpA vs. nr-axSpA), depending on the presence or absence of definite radiographic sacroiliac changes according to the modified New York criteria [2], respectively [3]. There is ample evidence that, from a clinical point of view, patients with nr-axSpA have a similar disease burden when compared with patients with r-axSpA [4–9]. The latter is, however, associated with a higher propensity to osteoproliferation potentially leading to spinal ankylosis and a greater impairment of spinal mobility [10, 11]. While a male predominance is found in r-axSpA, an equal male to female distribution has repeatedly been reported for nr-axSpA. Some important differences in clinical manifestations and response to treatment between the sexes have been delineated for the radiographic disease form [12–19]. In comparison to men, women with r-axSpA present with higher self-reported disease activity and functional impairment, a lower quality of life, less severe spinal radiographic changes, and more peripheral disease (arthritis and enthesitis). Objective markers of inflammation, such as elevated C-reactive protein (CRP) levels and magnetic resonance imaging (MRI) inflammation of the axial skeleton are more often found in men. It remains unclear, whether comparable differences between the sexes exist for nr-axSpA. Indeed, available data on gender differences in nr-axSpA is limited to subgroups (e.g., clinical arm of the ASAS classification criteria) [20]. Following the demonstration of response to tumor necrosis factor inhibitors (TNFi) also in active nr-axSpA following an inadequate response to nonsteroidal anti-inflammatory drugs (NSAIDs) [21–24], this subgroup of patients has been included in recent recommendations for the treatment of axSpA with biologic disease-modifying anti-rheumatic drugs (bDMARDs) [25, 26]. There exists some evidence for a differential effectiveness of treatment in female versus male patients with nr-axSpA [8, 27]. The aim of this study was to compare male and female patients classified as having nr-axSpA with regard to demographics, clinical manifestations and response to tumor necrosis factor inhibitors (TNFi) in a large national observational cohort.

## Patients and methods

### Study population

Patients with a clinical diagnosis of axSpA in the Swiss Clinical Quality Management (SCQM) cohort [6] were included in the current study if they met the following conditions: (a) fulfillment of the Assessment of SpondyloArthritis international Society (ASAS) classification criteria for axSpA [1] and (b) lack of definite radiographic sacroiliac changes according to the modified New York criteria [2]. The latter was assessed on collected radiographs of the pelvis, which were centrally digitized, and independently scored in a blinded matter by 2 rotating members of the SCQM axSpA scientific board (a total of 6 calibrated readers) [6]. Discrepancies between 2 readers in the evaluation of the sacroiliac joints were solved by consensus. Data on sacroiliac or spinal magnetic resonance imaging (MRI) scan positivity for inflammation was entered in the online database by the treating rheumatologist at each visit (expert opinion of local rheumatologist or radiologist; no central MRI scoring performed, as MRI scans were not collected).

Clinical evaluations were recommended at inclusion in the cohort, at yearly intervals thereafter, as well as at treatment changes and were performed according to the recommendations of ASAS [28]. Enthesitis was assessed with the Maastricht Ankylosing Spondylitis Enthesitis Score (MASES), modified to include the proximal insertion of the plantar fasciae. Rheumatologists indicate the presence of enthesitis by clicking on the specified enthesitis location on a homunculus shown from front and back. The rheumatologist is reminded of the intensity of the pressure to test for enthesitis with a written note adjacent to the homunculus: “thumb pressure of approximately 4 kg, corresponding to the pressure leading to discoloration of a third of the thumbnail.”

The diagnosis of concurrent fibromyalgia was based on the expert opinion of the treating rheumatologist and reported through a comorbidity questionnaire. The rheumatologist is automatically led through the comorbidity questionnaire in the online database at the time of reporting the results of the clinical examination to ensure systematic completion of the form. The proportion of the population with available data on comorbidities was 62%. The fulfillment of classification criteria for fibromyalgia was not required and no standardized fibromyalgia questionnaire was used.

Data on patients recruited into SCQM from January 2005 to November 2018 were available for the current analysis. The Ethics committee of the Canton of Zurich (KEK-ZH-Nr. 2014-0439) approved the study and written informed consent was obtained from all patients prior to inclusion into SCQM.

### Treatment effectiveness

Assessment of response to treatment with a first TNFi was analyzed in patients with available information on disease activity at start of treatment, as well as with an available outcome at 1 year (± 6 months). Patients with concomitant fibromyalgia were excluded from the analyses if this feature was reported either at baseline or at any time-point during follow-up. Given the fact the patients treated with biologics are preferentially included in SCQM, we expected a lower proportion of patients with fibromyalgia in comparison to other studies, assuming that rheumatologists might be reluctant to install biologics in axSpA patients diagnosed with concurrent fibromyalgia.

Response was either assessed in patients still on treatment at this time-point (completer analysis) or in all patients—with those having discontinued treatment in the meantime being considered non-responders (response/tolerance analysis) [18]. The achievement of the 40% improvement ASAS criteria (ASAS40) was considered as the primary outcome. We also analyzed additional response rates: ASAS20 response criteria, the proportion of patients achieving an Ankylosing Spondylitis Disease Activity Score (ASDAS) < 2.1 or an ASDAS < 1.3 (remission), as well as a clinically important and major improvements in ASDAS. It is important to note that the majority of TNFi (adalimumab, etanercept, golimumab and infliximab) are approved for the treatment of “Bechterew’s” disease after inadequate response to conventional treatment in Switzerland, with no differentiation being made in the label between the radiographic and the nonradiographic disease form. The label for certolizumab-pegol differs in this respect. It is approved in Switzerland for the treatment of severe active axial spondyloarthritis, including patients with severe active ankylosing spondylitis as well as severe active nr-axSpA, if the patients have inadequately responded to conventional treatment or had experienced adverse events to nonsteroidal anti-rheumatic drugs. Moreover, patients with severe active nr-axSpA requiring certolizumab-pegol should present with objective signs of inflammation (either on MRI or an elevated C-reactive protein (CRP)).

### Statistical analyses

Comparisons between baseline characteristics in women and men were performed using the Fisher’s exact test for categorical variables and the Mann-Whitney *U* test for continuous variables. The Fisher’s exact test was used to assess the significance of differences in response rates between genders. An adjusted odds ratio for different response criteria was estimated through logistic regression analysis. The following variables were included in the models as they differed between men and women at start of treatment and could potentially be mediators of the effect of sex on treatment response: diagnostic delay, degree of enthesitis, the level of the Bath Ankylosing Spondylits Disease Activity Index (BASDAI), and the body mass index (BMI). Drug retention was assessed with Kaplan-Meier plots and differences in retention rates were analyzed with the log-rank test. All tests were 2-sided (significance level set at 0.05). R statistical software was utilized for all analyses.

## Results

### Baseline characteristics

From a total of 1818 patients fulfilling the ASAS classification criteria with an available baseline pelvis radiograph, 495 patients presented without definite sacroiliac changes according to central scoring and were included as nr-axSpA in the current study (231 men and 264 women). Baseline characteristics of these patients are shown in Table 1 (A). Women were slightly older at inclusion, which was mainly due to a significantly longer diagnostic delay, as the age at symptom onset was comparable between the genders. In comparison to men, a slightly lower proportion of women was HLA-B27 positive (67.0% vs. 76.5%, *p* = 0.03). Mean ± SD disease activity as assessed by the BASDAI was higher in women than in men (5.3 ± 2.1 vs. 4.6 ± 2.2, *p* = 0.003). CRP levels and the proportion of patients with elevated CRP were comparable between the sexes, as was the ASDAS. Moreover, there were no differences with regards to impairment of physical function (BASFI), spinal mobility (BASMI), and of quality of life (EQ-5D). Regarding peripheral manifestations, the proportion of men and women with peripheral arthritis and dactylitis was comparable. However, female nr-axSpA patients presented more often with peripheral enthesitis and with a higher MASES. The proportion of patients with concomitant clinically diagnosed fibromyalgia was higher in women than in men (13.1% vs. 2.7%, *p* < 0.001). After excluding patients with diagnosed concurrent fibromyalgia from the analyses, remaining gender differences in BASDAI were mainly due to fatigue and enthesitis, both more prominent in women than in men (Table 1 (B)). Further comparisons did not reveal any sex differences with regards to extra-skeletal manifestations (uveitis, psoriasis, or inflammatory bowel disease), smoking status, education, and work absenteeism. The only additional difference between the sexes concerned the BMI (24.1 vs. 25.6 on average for women and men, respectively).

**Table 1:** Characteristics of all patients classified as having nonradiographic axial spondyloarthritis at inclusion in SCQM

Parameter	A. All nr-axSpA patients				B. All nr-axSpA patients excluding patients with co-morbid FM			
	***N, 495***	Men, ***N*** = 231	Women, ***N*** = 264	***p***	***N, 470***	Men, ***N*** = 227	Women, ***N*** = 243	***p***
Age, years	495	36.6 (10.9)	38.2 (10.7)	0.09	470	36.4 (10.9)	38.1 (10.7)	0.09
Age at symptom onset, years	481	28.3 (8.4)	28.7 (9.1)	0.65	456	28.2 (8.4)	28.6 (9.0)	0.65
Symptom duration, years	481	8.3 (9.2)	9.6 (9.8)	0.10	456	8.2 (9.2)	9.5 (9.8)	0.12
Diagnostic delay, years	**480**	**4.7 (7.6)**	**6.0 (7.8)**	**0.005**	**455**	**4.7 (7.5)**	**6.0 (7.8)**	**0.01**
HLA-B27 positive, %	**453**	**76.5**	**67.0**	**0.03**	**430**	**77.0**	**68.2**	**0.05**
Positive family history for spondyloarthritis, %	417	32.0	40.5	0.08	**393**	**32.1**	**42.0**	**0.05**
Prior sacroiliitis on MRI, %	477	52.9	58.7	0.23	453	53.9	58.5	0.34
BASDAI	**430**	**4.6 (2.2)**	**5.3 (2.1)**	**0.003**	**412**	**4.6 (2.2)**	**5.1 (2.1)**	**0.02**
• BASDAI 1 (fatigue)	**436**	**4.7 (2.8)**	**6.0 (2.4)**	**< 0.001**	**418**	**4.7 (2.8)**	**5.9 (2.4)**	**< 0.001**
• BASDAI 2 (back pain)	**436**	**5.9 (2.7)**	**6.5 (2.5)**	**0.03**	418	5.9 (2.7)	6.3 (2.5)	0.10
• BASDAI 3 (joint pain/swelling)	**435**	**3.6 (3.1)**	**4.2 (3.1)**	**0.04**	417	3.6 (3.1)	4.1 (3.1)	0.07
• BASDAI 4 (enthesitis)	**433**	**4.2 (3.3)**	**5.0 (3.1)**	**0.02**	**415**	**4.2 (3.3)**	**4.8 (3.0)**	**0.04**
• BASDAI 5 (intensity of morning stiffness)	434	5.2 (3.0)	5.4 (3.1)	0.65	416	5.3 (3.0)	5.2 (3.1)	0.96
• BASDAI 6 (duration of morning stiffness)	433	4.0 (2.9)	4.1 (2.9)	0.68	415	4.0 (2.9)	4.0 (2.9)	0.99
Physician Global Assessment	481	3.7 (2.2)	3.8 (2.0)	0.49	412	3.7 (2.2)	3.7 (2.0)	0.86
Patient Global Assessment	430	5.3 (2.9)	5.6 (2.8)	0.27	412	5.3 (2.9)	5.5 (2.8)	0.54
ASDAS	402	2.9 (1.0)	2.9 (0.9)	0.23	386	2.9 (1.0)	2.9 (0.9)	0.49
CRP (mg/l), median (IQR)	463	4.0 (1.0; 8.0)	4.0 (2.0; 8.0)	0.86	441	4.0 (1.1; 8.0)	4.0 (2.0; 8.0)	0.96
Elevated CRP, %	459	26.5	26.6	1.00	437	26.5	26.1	1.00
BASFI	435	2.8 (2.4)	3.1 (2.5)	0.16	416	2.8 (2.4)	2.9 (2.4)	0.54
BASMI	473	1.3 (1.4)	1.3 (1.3)	0.98	449	1.4 (1.4)	1.3 (1.2)	0.72
EQ-5D	431	61.4 (22.5)	59.7 (20.1)	0.34	412	61.3 (22.6)	61.2 (19.4)	0.78
IBP according to ASAS, %	462	80.5	77.3	0.43	437	80.6	77.0	0.41
Spinal MRI inflammation, %	476	26.9	24.9	0.67	452	26.9	23.6	0.45
Spinal radiographic changes, %	476	6.3	3.6	0.20	452	5.9	3.0	0.17
Current peripheral arthritis,%	489	35.8	39.2	0.46	464	36.0	38.9	0.57
Number of swollen joints	481	0.7 (1.9)	0.9 (2.3)	0.38	457	0.7 (1.9)	0.7 (1.8)	0.54
Current enthesitis, %	**483**	**64.0**	**79.6**	**< 0.001**	**459**	**63.8**	**78.3**	**< 0.001**
Modified MASES	**481**	**1.9 (2.5)**	**3.2 (3.4)**	**< 0.001**	**457**	**1.8 (2.5)**	**2.9 (3.1)**	**< 0.001**
Co-morbid fibromyalgia, %	**307**	**2.7**	**13.1**	**< 0.001**	–	–	–	–
Dactylitis ever, %	492	10.9	11.4	0.89	468	10.6	12.0	0.66
Uveitis ever, %	434	18.1	13.1	0.18	410	18.4	12.9	0.14
Psoriasis ever, %	373	9.1	9.1	1.00	351	9.3	8.4	0.85
Inflammatory bowel disease ever, %	427	6.0	7.9	0.46	403	5.6	8.2	0.33
Taking NSAIDs, %	471	87.3	90.4	0.31	447	87.6	89.6	0.55
Taking methotrexate, %	**495**	**5.2**	**10.2**	**0.04**	470	5.3	9.9	0.08
Taking sulfasalazine, %	495	4.8	6.1	0.56	470	4.8	6.2	0.55
Taking TNFi, %	495	14.7	15.5	0.90	470	14.5	15.2	0.90
Ever TNFi, %	495	68.0	72.0	0.78	470	67.4	70.8	0.80
Current smoking, %	432	32.0	30.2	0.75	413	32.0	28.3	0.45
Body mass index	**483**	**25.7 (3.9)**	**24.3 (4.5)**	**< 0.001**	**458**	**25.6 (3.9)**	**24.1 (4.2)**	**< 0.001**
Education, %	462			0.46	440			0.52
• Compulsory		15.8	19.8			15.2	18.3	
• Vocational		56.7	51.8			57.4	52.4	
• University		27.4	28.3			27.5	29.3	
Absenteeism within last year, %	389	53.9	44.0	0.12	372	53.9	43.2	0.10

All patients with nr-axSpA are presented in A, while patients with co-morbid fibromyalgia are excluded in B. Except where indicated otherwise, values are the mean (SD). Data in bold are statistically significant. *ASAS* Assessment of SpondyloArthritis international Society, *ASDAS* Ankylosing Spondylitis Disease Activity Score, *BASDAI* Bath Ankylosing Spondylitis Disease Activity Index, *BASFI* Bath Ankylosing Spondylitis Functional Index, *BASMI* Bath Ankylosing Spondylitis Metrology Index, *CRP* C-reactive protein, *EQ-5D* EuroQol 5-domains, *FM* fibromyalgia, *HLA-B27* human leucocyte antigen B27, *IBP* inflammatory back pain, *MASES* Maastricht Ankylosing Spondylitis Enthesitis Score, modification refers to the inclusion of the plantar fascia in the count, *MRI* magnetic resonance imaging, *nr-axSpA* nonradiographic axial spondyloarthritis, *NSAIDs* nonsteroidal anti-inflammatory drugs, *TNFi* tumor necrosis factor inhibitor

### Response to TNFi treatment

Treatment response was investigated in nr-axSpA patients without concomitant fibromyalgia. A first TNFi was initiated in 163 patients (Table 2). Corresponding to sex differences seen in the whole nr-axSpA cohort, women starting treatment had a longer diagnostic delay, higher BASDAI and MASES, and a lower BMI in comparison to men. The proportion of women initiating TNFi at an ASDAS level > 2.1 was 96.2%, while it was only 83.9% in men (*p* = 0.02). A follow-up visit at 1 year to assess treatment response was available in 120 patients (73.6%) and in a comparable proportion of women vs. men (72.9% vs. 74.4%, respectively). Baseline characteristics of these patients (Table 3) were similar to the findings in all nr-axSpA patients starting a TNFi (Table 2). In response/tolerance analyses, an ASAS40 response was achieved by 17% of women and 38% of men (OR 0.34; 95% CI 0.12, 0.93; Table 4). ASAS40 responses were even lower in women than in men after adjustment for baseline differences in BASDAI, MASES, BMI and diagnostic delay (OR 0.19; 95% CI 0.05, 0.61; Tables 4 and 5). A higher BMI was associated with a lower ASAS40 response (Table 5). In contrast, higher BASDAI levels were associated with a trend for better ASAS40 response. Crude response analyses and adjusted analyses for other outcomes confirmed these results (Table 4). Comparable results were obtained in a sensitivity analysis of the response/tolerance analysis, excluding patients having stopped the TNFi because of other reasons for discontinuation and imputing patients having discontinued the TNFi due to remission as responders (Table 6). All adjusted treatment responses were still significantly lower in women in comparison to men in nr-axSpA patients still on first TNFi at 1 year (completer analysis; OR 0.25; 95% CI 0.06, 0.91; Tables 4 and 5).

**Table 2:** Characteristics of nr-axSpA patients at initiation of first TNFi treatment after exclusion of patients with co-morbid fibromyalgia

Parameter	***N***, 163	Men, ***N*** = 78	Women, ***N*** = 85	***p***
Age, years	163	35.6 (10.8)	39.1 (11.4)	0.10
Age at onset, years	162	27.9 (8.6)	28.1 (8.5)	0.66
Symptom duration, years	**163**	**7.6 (8.8)**	**10.9 (10.8)**	**0.05**
Diagnostic delay, years	**162**	**4.1 (7.6)**	**7.8 (9.9)**	**0.005**
HLA-B27 positive, %	149	75.7	68.0	0.36
Prior sacroiliitis on MRI, %	154	70.8	68.3	0.86
BASDAI	**148**	**5.3 (2.0)**	**6.3 (1.6)**	**0.003**
Physician Global Assessment	155	5.0 (1.9)	4.8 (1.6)	0.63
Patient Global Assessment	149	6.4 (2.2)	6.7 (2.1)	0.30
ASDAS	140	3.3 (1.0)	3.4 (0.7)	0.29
ASDAS > 2.1, %	**140**	**83.9**	**96.2**	**0.02**
CRP (mg/l), median (IQR)	154	6.0 (2.0; 12.0)	5.0 (3.0; 9.0)	0.43
Elevated CRP, %	154	42.5	38.3	0.62
BASFI	148	3.6 (2.4)	3.8 (2.5)	0.54
BASMI	141	1.2 (1.1)	1.4 (1.2)	0.42
EQ-5D	141	54.8 (22.8)	55.8 (18.3)	0.79
Current periph. Arthritis,%	159	41.6	52.4	0.20
Number of swollen joints	155	0.7 (1.2)	1.3 (2.5)	0.32
Current enthesitis, %	158	80.5	85.2	0.53
Modified MASES	**157**	**2.3 (2.5)**	**3.9 (3.3)**	**0.002**
Dactylitis ever, %	161	13.2	21.2	0.21
csDMARDs ever, %	163	26.9	41.2	0.07
Taking NSAIDs, %	150	92.9	86.2	0.29
Current smoking, %	139	28.3	22.8	0.55
Body mass index	**160**	**25.9 (4.2)**	**24.0 (4.4)**	**< 0.001**
Type of TNFi, %	163			0.25
• Adalimumab		34.6	45.9	
• Certolizumab		1.3	0.0	
• Etanercept		19.2	15.3	
• Golimumab		20.5	24.7	
• Infliximab		24.4	14.1	

Except where indicated otherwise, values are the mean (SD). Data in bold are statistically significant. Patients with co-morbid fibromyalgia were excluded from these analyses. *ASDAS* Ankylosing Spondylitis Disease Activity Score, *BASDAI* Bath Ankylosing Spondylitis Disease Activity Index, *BASFI* Bath Ankylosing Spondylitis Functional Index, *BASMI* Bath Ankylosing Spondylitis Metrology Index, *CRP* C-reactive protein levels; *csDMARDs* conventional synthetic disease-modifying anti-rheumatic drugs, *EQ-5D* EuroQol 5-domains, *HLA-B27* human leucocyte antigen B27, *MASES* Maastricht Ankylosing Spondylitis Enthesitis Score, modification refers to the inclusion of the plantar fascia in the count, *nr-axSpA* nonradiographic axial spondyloarthritis, *NSAIDs* nonsteroidal anti-inflammatory drugs, *TNFi* tumor necrosis factor inhibitor

**Table 3:** Characteristics of nr-axSpA patients at initiation of first TNFi treatment with available follow-up visit at 1 year (patients with co-morbid fibromyalgia excluded)

Parameter	A. Nr-axSpA patients with available follow-up visit at 1 year				B. Nr-axSpA patients still on TNFi treatment at 1 year			
	***N, 120***	Men***, N*** = 58	Women***, N*** = 62	***p***	***N, 83***	Men***, N*** = 45	Women***, N*** = 38	***p***
Age, years	120	36.0 (10.8)	38.6 (11.4)	0.25	83	34.6 (10.3)	39.2 (13.4)	0.16
Age at onset, years	120	27.7 (8.4)	27.9 (8.2)	0.78	83	27.4 (8.0)	27.4 (7.5)	0.89
Symptom duration, years	120	8.3 (9.5)	10.7 (11.3)	0.27	83	7.2 (7.8)	11.7 (12.2)	0.12
Diagnostic delay, years	**120**	**4.7 (8.6)**	**7.3 (10.1)**	**0.03**	**83**	**3.0 (5.3)**	**7.4 (10.3)**	**0.02**
HLA-B27 positive, %	110	79.6	73.2	0.50	78	83.3	77.8	0.58
Prior sacroiliitis on MRI, %	112	67.9	67.8	1.00	77	66.7	65.7	1.00
BASDAI	**108**	**5.4 (2.0)**	**6.1 (1.6)**	**0.05**	74	5.2 (2.0)	5.9 (1.6)	0.09
Physician Global Assessment	115	5.1 (1.9)	4.8 (1.7)	0.54	81	5.3 (2.0)	4.7 (1.8)	0.28
Patient Global Assessment	109	6.6 (2.2)	6.7 (1.9)	1.00	75	6.5 (2.2)	6.4 (2.0)	0.89
ASDAS	100	3.4 (0.9)	3.4 (0.7)	0.63	69	3.4 (0.9)	3.4 (0.6)	0.90
ASDAS > 2.1, %	100	88.9	94.5	0.46	69	91.7	97.0	0.62
CRP (mg/l), median (IQR)	111	7.0 (2.0; 15.0)	7.0 (3.0; 10.0)	0.63	77	7.3 (3.0; 19.5)	7.0 (3.5; 10.5)	0.37
Elevated CRP, %	111	47.2	43.1	0.71	77	54.8	42.9	0.36
BASFI	109	3.7 (2.4)	3.5 (2.4)	0.61	75	3.5 (2.4)	3.2 (2.3)	0.53
BASMI	102	1.2 (1.1)	1.4 (1.2)	0.37	70	1.1 (1.1)	1.2 (1.3)	0.88
EQ-5D	103	53.7 (22.8)	57.6 (17.8)	0.52	70	55.6 (21.7)	59.2 (15.7)	0.65
Current periph. arthritis,%	116	47.4	47,5	1.00	82	46.7	54.0	0.66
Number of swollen joints	113	0.7 (1.2)	1.2 (2.6)	0.83	80	0.8 (1.3)	1.5 (2.9)	0.52
Current enthesitis, %	115	80.7	86.2	0.46	82	80.0	86.5	0.56
Modified MASES	**114**	**2.4 (2.6)**	**3.7 (3.2)**	**0.02**	**81**	**2.1 (2.2)**	**3.4 (2.7)**	**0.03**
Dactylitis ever, %	118	16.1	19.4	0.81	82	11.4	21.1	0.36
csDMARDs ever, %	120	31.0	40.3	0.34	83	26.7	36.8	0.35
Taking NSAIDs, %	109	94.1	86.2	0.21	**76**	**100.0**	**85.7**	**0.02**
Current smoking, %	102	28.9	15.8	0.15	68	26.5	8.8	0.11
Body mass index	**120**	**25.9 (4.2)**	**24.0 (4.4)**	**0.002**	**83**	**25.6 (4.2)**	**23.6 (4.7)**	**0.004**
Type of TNFi, %	120			0.69	83			0.38
• Adalimumab		34.5	43.5			35.6	50.0	
• Certolizumab		1.7	0.0			2.2	0.0	
• Etanercept		19.0	16.1			20.0	10.5	
• Golimumab		22.4	24.2			20.0	26.3	
• Infliximab		22.4	16.1			22.2	13.2	

A is the characteristics of patients starting TNFi included in the response/tolerance analyses: response in patients with available outcome at 1 year, patients having discontinued the first TNFi in the meantime being considered non-responders. B is the characteristics of patients starting TNFi included in the completer analyses at 1 year. Except where indicated otherwise, values are the mean (SD). Data in bold are statistically significant. Patients with co-morbid fibromyalgia were excluded from these analyses. *ASDAS* Ankylosing Spondylitis Disease Activity Score, *BASDAI* Bath Ankylosing Spondylitis Disease Activity Index, *BASFI* Bath Ankylosing Spondylitis Functional Index, *BASMI* Bath Ankylosing Spondylitis Metrology Index, *CRP* C-reactive protein levels, *csDMARDs* conventional synthetic disease-modifying anti-rheumatic drugs, *EQ-5D* EuroQol 5-domains, *HLA-B27* human leucocyte antigen B27, *MASES* Maastricht Ankylosing Spondylitis Enthesitis Score, modification refers to the inclusion of the plantar fascia in the count, *nr-axSpA* nonradiographic axial spondyloarthritis, *NSAIDs* nonsteroidal anti-inflammatory drugs, *TNFi* tumor necrosis factor inhibitor

**Table 4:** Clinical outcome of women versus men with nr-axSpA after 1 year of treatment with a first TNF inhibitor

Type of analysis	Outcome	Unadjusted analyses						Adjusted analyses^**#**^			
		***N***	Women %	Men %	OR	95% CI	***p***	***N***	OR	95% CI	***p***
Response/tolerance*	**ASAS20**	99	27	57	0.28	0.11; 0.68	0.002	93	0.16	0.04; 0.50	0.003
	**ASAS40**	99	17	38	0.34	0.12; 0.93	0.02	93	0.19	0.05; 0.62	0.009
	**BASDAI50**	98	23	50	0.30	0.11; 0.77	0.007	93	0.19	0.05; 0.58	0.005
	**ASDAS improvement ≥ 1.1**	84	28	58	0.29	0.10; 0.78	0.008	82	0.26	0.08; 0.75	0.02
	**ASDAS < 2.1**	93	27	49	0.39	0.15; 1.00	0.03	86	0.18	0.04; 0.65	0.01
	**ASDAS improvement ≥ 2**	84	4	26	0.13	0.01; 0.68	0.005	82	0.04	0.00; 0.27	0.003
	**ASDAS < 1.3**	93	8	29	0.23	0.05; 0.82	0.01	86	0.07	0.01; 0.39	0.005
Completer**	**ASAS20**	68	45	73	0.31	0.10; 0.94	0.03	66	0.15	0.03; 0.60	0.01
	**ASAS40**	68	29	49	0.44	0.14; 1.31	0.14	66	0.25	0.06; 0.91	0.04
	**BASDAI50**	67	39	64	0.36	0.12; 1.07	0.05	66	0.20	0.05; 0.75	0.02
	**ASDAS improvement ≥ 1.1**	61	46	67	0.44	0.14; 1.38	0.13	60	0.36	0.09; 1.29	0.13
	**ASDAS < 2.1**	67	43	59	0.53	0.18; 1.54	0.22	63	0.28	0.06; 1.12	0.08
	**ASDAS improvement ≥ 2**	61	7	30	0.18	0.02; 0.98	0.03	60	0.05	0.00; 0.38	0.01
	**ASDAS < 1.3**	67	13	35	0.29	0.06; 1.11	0.05	63	0.08	0.01; 0.47	0.01

*Response in patients with available outcome at 1 year, patients having discontinued the first TNFi in the meantime being considered non-responders. **Response in patients still on their first TNFi at 1 year. Patients with co-morbid fibromyalgia were excluded from these analyses. ^#^Adjustment for diagnostic delay, MASES, BASDAI and BMI. *ASAS20* and *ASAS40* 20% and 40% improvement according to the ASAS criteria, respectively; *ASDAS* Ankylosing Spondylitis Disease Activity Score; *BASDAI50* 50% improvement in the BASDAI; *nr-axSpA* nonradiographic axial spondyloarthritis; *TNF* tumor necrosis factor

**Table 5:** Adjusted ASAS40 response of women versus men after 1 year of treatment with a first TNF inhibitor

Variable	A. Response/tolerance analysis			B. Completer analysis		
	OR	95% CI	***p***	OR	95% CI	***p***
**Female vs. male**	0.19	0.05; 0.62	0.009	0.25	0.06; 0.91	0.04
**Diagnostic delay**	0.97	0.91; 1.03	0.41	0.98	0.90; 1.05	0.66
**Modified MASES**	0.82	0.61; 1.05	0.14	0.77	0.56; 1.02	0.08
**BASDAI**	1.24	0.91; 1.75	0.18	1.41	1.00; 2.08	0.06
**BMI**	0.78	0.64; 0.92	0.008	0.82	0.66; 0.97	0.04

A is the ASAS40 response in patients with available outcome at 1 year, patients having discontinued the first TNFi in the meantime being considered non-responders. B is the ASAS40 response in patients still treated with the first TNF inhibitor at 1 year. Patients with co-morbid fibromyalgia were excluded from both analyses. *ASAS40* 40% improvement according to the Assessment in SpondyloArthritis International Society criteria, *BASDAI* Bath Ankylosing Spondylitis Disease Activity Index, *BMI* body mass index; *CI* confidence interval; *MASES* Maastricht Ankylosing Spondylitis Enthesitis Score, modification refers to the inclusion of the plantar fascia in the count, *OR* odds ratio

**Table 6:** Sensitivity analysis: response/tolerance analysis at 1 year excluding patients having stopped TNFi due to other reasons than ineffectiveness and adverse events and imputing patients having discontinued the TNFi because of remission as being responders

Outcome	***N***	OR	95% CI	***p***
**ASAS20**	89	0.20	0.06; 0.62	0.008
**ASAS40**	89	0.25	0.07; 0.78	0.02
**BASDAI50**	89	0.24	0.07; 0.72	0.01
**ASDAS improvement ≥ 1.1**	78	0.32	0.10; 0.93	0.04
**ASDAS < 2.1**	82	0.26	0.06; 0.91	0.04
**ASDAS improvement ≥ 2**	78	0.08	0.01; 0.43	0.006
**ASDAS < 1.3**	82	0.14	0.02; 0.62	0.02

Analyses are adjusted for diagnostic delay, MASES, BASDAI, and BMI. *ASAS20* and *ASAS40* 20% and 40% improvement according to the ASAS criteria, respectively; *ASDAS* Ankylosing Spondylitis Disease Activity Score; *BASDAI50* 50% improvement in the BASDAI; *nr-axSpA* nonradiographic axial spondyloarthritis; *TNF* tumor necrosis factor

## Discussion

This real-life cohort of patients with nr-axSpA, with classification performed after central reading of pelvis radiographs, reveals less differences in demographics and clinical manifestations between women and men than previously found for the radiographic subgroup of axSpA. Despite continuous efforts for early diagnosis, diagnostic delay is, however, still considerable in nr-axSpA (approximately 5 years) and significantly longer (> 1 year) in women than in men. In contrast to r-axSpA, age at symptom onset in female vs. male patients is comparable in nr-axSpA [29]. Importantly, in contrast to r-axSpA, there were no differences between the genders with regard to important clinical parameters: disease activity as assessed by the ASDAS; the proportion of patients with elevated CRP; the level of CRP elevation; the proportion of patients with sacroiliac joint and spinal MRI inflammation; the presence of peripheral arthritis and dactylitis; functional and mobility impairments as assessed by the BASFI and BASMI, respectively; health-related quality of life; and global assessment of disease by patients or their rheumatologists. Despite these similarities between the genders in nr-axSpA, response to TNFi was significantly lower in women than in men (ASAS40 response: OR 0.19; 95% CI 0.05, 0.62; *p* = 0.009). This differences in ASAS40 response between the genders is greater than the one found in r-axSpA in our previous report (OR 0.44; 95% CI 0.21, 0.91; *p* = 0.03) [18]. The most important difference between men and women in nr-axSpA related to the BASDAI, as a patient-reported disease activity outcome. The amplitude of this difference was slightly lowered by exclusion of nr-axSpA patients with co-morbid fibromyalgia, which was more commonly seen in women, confirming previous analyses [30–32]. We cannot entirely exclude the possibility that diagnosis of concurrent fibromyalgia might have been missed in some patients, as clinical differentiation between enthesitis and allodynia of fibromyalgia might be challenging in clinical practice. However, our results were adjusted for the remaining differences in BASDAI and MASES. Moreover, a higher prevalence of enthesitis in women compared to men has consistently been shown in the radiographic disease state, not excluding a potential phenotypic distinction between the genders [33].

Less than 10% of women reached strict treatment response criteria, such as the ASDAS major improvement or remission criteria, while more than 25% of men with nr-axSpA achieved these responses. Male sex was also identified to be an important predictor of remission in nr-axSpA patients treated with adalimumab in the open-label phase of a recent trial [27], as well as in a Danish observational study investigating treatment response in AS vs. nr-axSpA after adjustment for sex [8]. Our current study therefore adds to available data to support the claim for future randomized controlled trials in axSpA to be sufficiently powered to detect potential sex differences [33].

Some limitations of our current analyses have to be acknowledged. Concern has arisen that since the introduction of the ASAS axSpA classification criteria misclassification or overtreatment might occur more frequently, particularly in nr-axSpA [34, 35]. There is increasing evidence that several imaging abnormalities might mimic mild sacroiliitis on MRI and some of these are more frequent in women (such as bone more edema following pregnancy or associated with osteitis condensans ilii) [36–38]. We were not able to evaluate the extent of this potential imaging misinterpretation, as MRIs were not available for central scoring. This also impeded the evaluation of potential sex differences regarding the extent and the intensity of sacroiliac joint inflammation in nr-axSpA [39, 40]. With regard to potential misdiagnosis of fibromyalgia as axSpA, it is important to note that patients with fibromyalgia rarely fulfill classification criteria for axSpA, as has recently been demonstrated [41, 42]. We have excluded axSpA patients with concurrent fibromyalgia from the treatment effectiveness analyses, if its presence was either reported at start of treatment or at any time-point during follow-up. However, we might have missed some patients, as the screening for fibromyalgia was based on the expert opinion of the treating rheumatologist reported on a comorbidity questionnaire and not through fulfillment of classification criteria for fibromyalgia or via the use of a standardized fibromyalgia questionnaire. The comorbidity form was available in 62% of the population as the rheumatologist was led through it in the online database before being able to enter the findings of the clinical examination to help with its systematic completion. Given the fact that patients treated with biologics are preferentially included in SCQM, we expected a lower proportion of patients with fibromyalgia in comparison to other axSpA studies, assuming that rheumatologists might be reluctant to install biologics in axSpA patients diagnosed with concurrent fibromyalgia.

We have found a lower proportion of HLA-B27 in women as compared to men, as has also been reported for the radiographic disease form in SCQM [18] as well as in the US PSOAS cohort [12]. While genotypic differences between genders cannot be entirely excluded, this finding might alternatively point to the possibility of a higher misclassification rate in women. We have not adjusted our response analyses for HLA-B27 as the respective proportions of positivity in men and women were comparable at start of treatment. This would indicate that rheumatologists might have been more confident of the axSpA diagnosis in women in the presence of HLA-B27 positivity in order to start TNFi treatment. The proportion of patients with a positive family history of spondyloarthritis was higher in women and this difference might potentially involve recall bias.

## Conclusion

Despite only few sex differences in baseline characteristics of patients with nr-axSpA, a disease subgroup known for its balanced sex ratio, response to treatment with TNFi was significantly lower in women than in men.

## Data Availability

The data sets analyzed during the current study are available from the corresponding author on reasonable request.

## Abbreviations

ASAS: Assessment in SpondyloArthritis international Society
ASAS40: 40% improvement in the ASAS criteria
ASDAS: Ankylosing Spondylitis Disease Activity Score
axSpA: Axial spondyloarthritis
BASDAI: Bath Ankylosing Spondylitis Disease Activity Index
BASFI: Bath Ankylosing Spondylitis Functional Index
BASMI: Bath Ankylosing Spondylitis Metrology Index
BMI: Body mass index
bDMARD: Biologic disease-modifying anti-rheumatic drug
CI: Confidence interval
CRP: C-reactive protein
HLA-B27: Human leucocyte antigen B27
MASES: Maastricht Ankylosing Spondylitis Enthesitis Score
MRI: Magnetic resonance imaging
nr-axSpA: Nonradiographic axial spondyloarthritis
NSAID: Nonsteroidal anti-inflammatory drug
OR: Odds ratio
r-axSpA: Radiographic axial spondyloarthritis
SCQM: Swiss Clinical Quality Management
TNFi: Tumor necrosis factor inhibitor
